# Endogenous Risk Factors of Cardiovascular Diseases (CVDs) in Military Professionals with a Special Emphasis on Military Pilots

**DOI:** 10.3390/jcm11154314

**Published:** 2022-07-25

**Authors:** Ewelina Maculewicz, Agata Pabin, Krzysztof Kowalczuk, Łukasz Dziuda, Agnieszka Białek

**Affiliations:** 1 Faculty of Physical Education, Jozef Pilsudski University of Physical Education in Warsaw, 00-968 Warsaw, Poland; ewelina.maculewicz@awf.edu.pl; 2 Military Institute of Aviation Medicine, 01-755 Warsaw, Poland; apabin@wiml.waw.pl (A.P.); kkowalczuk@wiml.waw.pl (K.K.); ldziuda@wiml.waw.pl (Ł.D.); 3 Department of Biotechnology and Nutrigenomics, Institute of Animal Genetics and Biotechnology of Polish Academy of Sciences, Postępu 36A Jastrzębiec, 05-552 Magdalenka, Poland

**Keywords:** atherosclerosis, crew members, diabetes, hyperlipidemia, hypertension, military pilots, obesity, oxidative stress, SNPs, soldiers

## Abstract

Cardiovascular disease (CVD) risk factors can be categorized as non-modifiable and modifiable. Modifiable risk factors include some exogenous and behavioral factors that can be easily modified, whereas endogenous modifiable risk factors, such as hypertension, hyperlipidemia, diabetes, or obesity may be modified to a limited extend. An increased prevalence of CVDs as well as their risk factors have been observed in military personnel, as specific military-related stressors are highly correlated with acute cardiac disorders. Military pilots are a subpopulation with great CVD risk due to an accumulation of different psychological and physical stressors also considered to be CVD risk factors. This review presents data concerning CVD risk in military professionals, with a special emphasis on military pilots and crew members. We also discuss the usefulness of novel indicators related to oxidative stress, inflammation, or hormonal status as well as genetic factors as markers of CVD risk. For a correct and early estimation of CVD risk in asymptomatic soldiers, especially if no environmental risk factors coexist, the scope of performed tests should be increased with novel biomarkers. An indication of risk group among military professional, especially military pilots, enables the implementation the early preventive activities, which will prolong their state of health and military suitability.

## 1. Introduction

The World Health Organization (WHO) defines cardiovascular diseases (CVDs) as a group of disorders of the heart and blood vessels, including coronary heart disease, cerebrovascular disease, peripheral arterial disease, rheumatic heart disease, congenital heart disease, deep vein thrombosis, and pulmonary embolism. CVDs nowadays are the leading cause of death globally. In 2019, about 17.9 million people died from CVDs, mainly from heart attack and stroke (85%), which are acute events caused by the obstruction of blood flow to the heart (hear attack) and brain (stroke) or by bleeding in the brain (stroke). CVDs are sometimes called ‘silent killers’, as frequently no characteristic symptoms accompany their development until the symptoms of heart attack or stroke appear [1]. However, different risk factors have been established as influencing the risk of CVDs. They can be divided into non-modifiable and modifiable (mainly behavioral) risk factors. Classic non-modifiable risk factors mainly include age, gender, ethnicity, and family history, but some studies also suggest genetic factors (especially the polymorphism of certain genes) as important risk factors of CVD development. Modifiable risk factors include some exogenous behavioral factors that can be easily modified, e.g., by the quitting of tobacco smoking, a reduction of salt consumption in the diet, an increase in fruit and vegetable dietary intake, regular physical activity, the cessation of harmful alcohol consumption, or stress avoidance. Endogenous modifiable risk factors, such as hypertension, hyperlipidemia, diabetes, and obesity, may be modified to a limited extent, as they partially result from aforementioned exogenous risk factors. Moreover, novel risk factors, including inflammation, non-alcoholic fatty liver disease (NAFLD), chronic kidney disease (CKD), systemic lupus erythematosus (SLE), rheumatoid arthritis (RA), inflammatory bowel disease (IBD), human immunodeficiency virus (HIV), thyroid disease, sex hormone levels and vitamin D levels, have also been suggested to influence CVD prevalence and development [2,3,4,5]. Apart from the rising prevalence of CVD risk factors, the appearance of subclinical atherosclerosis and the minimal effectiveness of prevention programs are recognized as the main reasons for high CVD mortality [6]. Sedentary occupations and sitting time also seem to promote CVDs, irrespective of physical activity levels. Time spent sedentary has an independent detrimental association with waist circumference, blood glucose, insulin, triglycerides, and high-density lipoproteins (HDL-C), which are CVD risk factors. Tigbe et al. showed that waist circumference (correlation coefficient, r = 0.28, *p* = 0.002), fasting triglycerides (*r =* 0.30, *p* = 0.002), and HDL-C (*r =* −0.38, *p* < 0.0001) were significantly and adversely associated with sedentary posture. Sedentary time was positively associated with coronary risk, as determined by the 10-year PROCAM risk (*r =* 0.33, *p* = 0.004) [7]. Moreover, Katzmarzyk et al., who evaluated sitting time and mortality in Canadians, revealed a progressively higher risk of CVD mortality (HR: 1.00, 1.01, 1.22, 1.47, 1.54; *p* for trend < 0.0001) increasing with sedentary time independent of leisure time physical activity [8].

## 2. CVD Risk in Cockpit Crew and Military Personnel

In aviation, the incidence rate, including both complete and partial incapacitations, ranges from 0.10 to 0.77 per million pilot flight hours in military services and commercial aviation operations [9]. In-flight incapacitation due to a medical cause is a rare event occurring up to 0.45 times per 106 flight hours or 0.25% per annum, and the rate of in-flight incapacitations increases with age [10]. Moreover, the report by Newman confirmed that the risk of a pilot suffering from an in-flight medical condition or incapacitation is low, but CVDs rank as the single biggest cause for medical disqualification of pilots, both military and civil, and continue to receive much research attention. The prevalence of in-flight pilot heart attacks was 0.005% of the total occurrences listed on the Australian Transport Safety Bureau database for the study period (1973–2006), but they accounted for 50% of the fatal accidents arising from medical or incapacitation events. Fatal accidents involved single-pilot operations, which is not surprising given that when the single pilot is incapacitated or dies in flight the chances of a fatal accident occurring rise considerably [11]. Cardiovascular impairments are the most important and serious contributing factors of sudden in-flight incapacitation, and 38% of sudden in-flight incapacitation incidents in airline pilots are caused by CVDs [9]. CVDs account for 50% of all pilot licenses declined or withdrawn for medical reasons in Western Europe [12]. Those CVDs related to the in-flight incapacitations of commercial pilots are sudden cardiac death, acute coronary syndrome, cardiac arrhythmias, pulmonary embolism, and stroke [10]. In military pilots, coronary artery disease is a significant cause of potential in-flight incapacitation and the most common reason for permanent flying incapacitation. Sudden cardiac death has been found to be the initial presentation of coronary artery disease in 20% to even 80% of military pilots [13]. 

Airline pilots and the cockpit crew seem to be a subpopulation that is especially prone to CVD appearance. These conditions comprise prolonged sedentary posture; difficulties in moving the limbs; occupational stress; emotional tension; highly demanding tasks, especially during take-off and landing; and the predominance of intellectual activity over physical activity. They also encompass forced operation speed against time shortage; frequent time-zone changes; night-time, meridional, and trans-meridional flights; acceleration; G-loads; noise; vibration; and an unbalanced diet high in saturated fat [14,15,16]. That is why CVD risk stratification has been applied by aviation authorities in many countries [4,17,18,19]. 

Investigations of CVD risk in airline pilots are not satisfyingly described in the literature [20], but Huster et al. mentioned the highest number of incapacitations per year in pilots aged 50–59 (3.97 for 50–54 and 3.43 for 55–59) due to increasing cardiovascular death risk [21]. Moreover, Stuck et al. confirmed that the risk of CVD incapacitation increased with age, but they emphasized the capability of early identification of pilots with non-acceptable CVD risk [22]. A systematic review by Huster et al. confirmed that incapacitation prevalence increases with age [21]. European rules dictate age limits of 60 years for commercial single-pilot operations and of 65 years for multi-pilot operations [10]. However, Blettner et al. observed low cardiovascular mortality in airline pilots and flight engineers and claimed that occupational risk factors seem to be of limited influence with regard to the mortality of cockpit crews in Europe [23]. This was confirmed by the retrospective study by Zeeb et al. in a German cockpit crew. They observed relatively low cardiovascular mortality (resulting mainly from low tobacco smoking prevalence and an early detection of CVD due to regular annual or semi-annual aeromedical investigations) and significantly lower CVD mortality risk in cockpit crew employed before age 30 [14]. Stavola et al. also confirmed lower CVD mortality in flight crew and air traffic control officers. They link this observation with regular medical examinations and a lower prevalence of smoking and other risk factors in this group. Moreover, they observed no influence of occupational exposures characteristic for flight crew and air traffic control officers [24]. Moreover, hypoxic conditions present a continual threat for pilots, even with pressurized aircraft cabins, but Sucipta et al. did not reveal any dependencies between the time of useful consciousness and cardiovascular endurance (VO_2max_) in Indonesian military pilots [25]. A recent systematic review by Wilson et al. revealed a substantial prevalence (>50%) for overweight and obesity, insufficient physical activity, elevated fatigue, and regular alcohol intake among pilots [26].

The Civil Aviation Authority of Malaysia decided that a return to flying duties for a civilian or private pilot, which is the reassessment and recertification, can be completed six months after the cardiovascular event. For a military pilot, however, this waiting period is extended to 1 year. The 4 × 4 aeromedical risk matrix can serve as a very useful tool that can aid in decision making for the assessment and recertification process. Recent studies by Mohammad et al. showed that after coronary artery disease a pilot must be hemodynamically stable with no evidence of significant inducible ischemic left ventricular dysfunction and a minimum of 50% of the ventricular ejection fraction (LVEF). Moreover, a follow-up is recommended at six months after recertification and then annually with a routine noninvasive cardiac assessment [27].

The results of some studies show that there is also an increased prevalence of CVD as well as its risk factors in military personnel, as specific military-related stressors are highly correlated with acute cardiac disorders [3]. Mirzaeipour et al. emphasized that the working and environmental conditions of military personnel are very different from those for ordinary people and these conditions can also affect the risk factors of CVDs [28]. Studies by Gielerak et al. revealed that CVD risk factors were present in over 50% of Polish soldiers, and exposure to extensive stress and threats in combat conditions was recognized as a specific military-related stressor of additive detrimental effect [6]. Nikolova et al. confirmed that military pilots are exposed to stress factors in the work environment, which affect autonomic cardiovascular control in them [29]. According to Florea et al., CVD risk factors in the active military population correlate with a type of activity in which the degree of stress is extremely high and associated with intense physical exercise, frequently changing climate and time zone conditions, the sense of imminent danger, sleep deprivation, and a frequently unbalanced diet [30].

Military aircrew are of special concern, as they are exposed to significant additional demands when conducting their duties. These demands may be due to the environmental factors associated with flying high-performance aircraft (such as hypoxia or sustained acceleration) and undertaking their activity in a hostile operational environment (from flying over enemy territory to being engaged in air combat maneuvers). They also could be related to merely operating from base environments that are not conducive to the usual maintenance of circadian rhythms or sleep patterns [12]. These psychological and physical stressors are also considered to be risk factors, and their accumulation may affect CVD risk in military pilots. Stoney et al. revealed in a study on airline pilots that both chronic and acute stressors significantly increased lipid parameters in comparison to no-stressor periods, but the patterns differed in chronic and acute stress conditions [31]. Moreover, modern fast military jet aircraft place significantly greater strain on the cardiovascular system than previous aircraft [12]. On the other hand, as military air pilots undergo a screening examination annually from their first medical evaluation, which determines the fitness of a candidate for a military pilot career, they constitute a convenient study group for CVD risk factor evaluation and CVD risk progression [18,19].

## 3. CVDs Risk Scores

For many years the “1% rule”, which states that no pilot should have an annual risk of cardiovascular incapacitation exceeding 1%, has served as a possible standard and is endorsed as the threshold of choice by the International Civil Aviation Organization (ICAO), but the methods of determining the risk to an individual pilot vary by jurisdiction [32]. The Framingham risk score (FRS) is a common and simple tool used for predicting of the risk of CVD development in next 10 years based on data related to age, gender, total cholesterol, status of diabetes, systolic blood pressure, high-density lipoprotein cholesterol (HDL), and tobacco smoking [3]. The FRS and the risk prediction charts or tables that were derived from the Framingham function are widely used internationally [33]. Studies by Parastouei et al. revealed that in Iran 96.5% of the investigated male military personnel had FRSs less than 10%, 2% of these people showed moderate risk (10–19%), and 1.5% had a risk above 20% [3]. Assmann et al. designed an improved point-scoring scheme based on the completed 10-year follow-up of the cohort of middle-aged men in PROCAM. The use of their scoring scheme resulted in very little loss of information and was associated with greater predictive power than similar schemes [34]. Wirawam et al. examined Oceania-based civil airline pilots with a modified version of the FRS, the 5-year cardiovascular (5-year CVD) risk score. Excessive 5-year CVD risk scores were revealed in *n* = 30 participants (of which, 25 pilots had 5-year CVD risk scores of 10–15% and 5 pilots had 5-year CVD risk scores of 15–20%). In the comparative group, pilots whose 5-year CVD risk scores were lower than 10% were included, which resulted in *n* = 595 pilots in this group. The authors assessed different CVD risk factors, both endogenous and exogenous, including age, smoking history, diabetes status, history of hypertension, systolic blood pressure, diastolic blood pressure, history of hyperlipidemia, HDL cholesterol, triglycerides, and the total cholesterol–HDL ratio. All investigated risk factors were found to be elevated in the high-risk group of pilots compared to those in lower risk group, with the most pronounced differences observed for hypertension appearance (36.7% versus 6.1%), hyperlipidemia (40.0% versus 10.4%), diabetes (10% versus 0.5%), and tobacco smoking (26.7% versus 13%). Among the investigated high-risk pilot group, nine abnormal resting ECG results were observed. Twenty-six pilots from the high-risk group underwent exercise stress ECGs. In three patients, the exercise stress ECGs were judged to be borderline, and the exercise stress ECG was judged to be positive in four cases. In total, coronary angiography was performed in nine patients from the high-risk group and revealed five patients who had lesions characterized by more than 70% stenosis or occlusions in one or more arteries that were considered significant. The authors emphasized that exercise electrocardiograms, as a diagnostic test to investigate excessive cardiovascular risk in pilots, are not adequate to detect disease or to protect pilots from unnecessary invasive procedures, such as coronary angiography. They suggest the inclusion of laboratory markers and the use of different noninvasive imaging to create a more comprehensive and accurate cardiac investigation algorithm of CVD risk assessment and CVD detection [20]. Wiraman et al. also revealed that more than half (60%) of the CVD events in airline pilots occurred as sudden clinical presentations, and almost half (47%) of them occurred in pilots whose highest 5-year CVD risk was in the 5–10% range [17]. This suggests that the FRS and its modified version (the 5-year CVD risk score) are not sufficient for CVD risk prediction in airline pilots. Although it is commonly used in asymptomatic patients and is also recommended by military authorities and aviation authorities in many countries, some of its limitations have been revealed. One of the alternative methods that is highly recommended by Wiraman et al. is the quantification of the coronary artery calcium score (CACS) using computed tomography (CT), which can improve risk prediction over conventional risk prediction models. The CACS may be applied for cardiovascular risk assessment in people at intermediate risk or 10–20% over 10 years, as it has a progressively higher level of quality evidence in its role in the risk stratification of asymptomatic populations. They revealed that the CACS correctly reclassified 39% of asymptomatic patients into low-, intermediate-, and high-risk categories. However, the examined patients were not airline pilots. Nevertheless, they suggest that the CACS should be employed in the CVD risk assessment algorithm in a specific occupational group such as airline pilots [17]. Apart from the CACS, the carotid intima–media thickness (cIMT) is also considered as a noninvasive modality that can be used as evidence of atherosclerosis in both symptomatic and asymptomatic individuals. Its utility for CVD prediction has been confirmed both in men and women in epidemiological studies [35]. A review by Wirawan et al. also confirmed that the CACS is the highly recommended system for CVD risk assessment in intermediate-risk (10-year CVD risk score of 10–20%) asymptomatic adults. Other tests, such as the measurement of carotid intima–media thickness, supplemented by carotid plaque and the ankle brachial index, may also be useful, especially for the prevention of peripheral artery disease and stroke. Moreover, a stress myocardial perfusion scan should be considered as a potential cardiac functional test to be used with pilots with 5-year risks > 15% [33]. This prompted Wirawan et al. to propose a new system of CVD risk assessment in civil airline pilots in New Zealand. It applies a 5-year CVD risk score using cardiovascular risk charts as the first step of the screening process, followed by a CACS in asymptomatic pilots with 5-year CVD risks of 5–10% and 10–15%. For pilots with CACS < 400, drug and/or lifestyle interventions are recommended, whereas for those with CACS > 400 stress, myocardial perfusion imaging or a coronary computed tomography angiography are recommended. For patients with normal result on those tests, similar lifestyle and/or drug interventions are foreseen. However, if some abnormal results are found, a coronary angiography is recommended for those pilots and, as a result, a definitive treatment such as percutaneous coronary intervention, if necessary [36]. Studies by Grosz et al. estimated the CVD risk in Hungarian military pilots and showed that the CVD risk calculated by the Futrex program was elevated in 40% of the study population (levels 3 and 4), but the 5-year risk of CVD was below 2.5% in 50% of the pilots and did not exceed 15 to 20%, even in the group with the highest risk. In military pilots over 45 years old, blood fat parameters and the overall risk of CVDs improved over time in comparison to their earlier values, which may result from the strict selection due to regular screening examinations and the changes of lifestyle due to the efforts undertaken to stay in service [19]. In population studies in the USA during a 10-year surveillance period, it was revealed that 18.1% of all military service members were diagnosed with at least one of the CVD risk factors, whereas at least one CVD was diagnosed in 0.6% of all military service members [4]. Some of them were endogenous CVD risk factors, hypertension, overweight or obesity, diabetes, and hyperlipidemia, that frequently coexist as metabolic syndrome. Gray et al. described the three-dimensional risk matrix approach, which considers the probability of a medical event, the severity of its outcome, and the operational role of the individual in question to stratify the allowable risk. By acknowledging that the allowable rate of medical events will not be equal for all individuals, all medical conditions, or all situations, the risk matrix approach serves as a procedural framework by which the allowable risk can be determined. This framework can be applied to a wide variety of clinical situations [37]. Mulloy and Wielgosz recently proposed the acceptable annual risk formula, which takes into consideration the unique variables inherent in flight for setting CVD risk in commercial pilots [32]. Moreover, Holdsworth et al. proposed a three-dimensional (3D) risk matrix for practical decision making, where the acceptable clinical event rate is very low, to be used within high-hazard occupational medicine. This matrix integrates (1) the annual likelihood of a clinical event, (2) the degree of medical incapacitation that will result, and (3) the impact of this combination, given the importance of the individual’s role [38]. In 2021, Davenport et al. proposed, based on published literature from 70 years of aircrew-specific cardiac data gathered from nearly 1.3 million studies performed on over 300,000 aircrew, a new evidence-based coronary artery disease screening algorithm that will decrease risk of short-term sudden incapacitation from cardiac etiology to <0.5% [39].

## 4. Endogenous CVD Risk Factors

### 4.1. Hypertension

According to the WHO, hypertension is diagnosed if, when it is measured on two different days, the systolic blood pressure (the pressure in blood vessels when the heart contracts) is ≥140 mmHg and/or the diastolic blood pressure (the pressure in the vessels when the heart rests between beats) is ≥90 mmHg [40]. The most important risk factors of hypertension are excessive salt consumption, high saturated and *trans* fatty acid dietary intake, a diet poor in fruits and vegetables, physical inactivity, tobacco smoking, alcohol consumption, and overweight or obesity, all of which are modifiable risk factors. Non-modifiable risk factors include a family history of hypertension, age over 65 years, and coexisting diseases such as diabetes or kidney disease [40]. It is clear that hypertension shares some of the non-modifiable and modifiable risk factors with CVDs, itself being, at the same time, a risk factor of CVDs. Moreover, raised blood pressure usually does not manifest with any specific symptoms, and it can develop for years, exerting an overall harmful impact on the organism. It is of utmost importance to measure blood pressure regularly, to diagnose elevated blood pressure as soon as possible, and to implement appropriate actions to prevent hypertension development. Excessive pressure in blood vessels can harden arteries, which can lead to decreases in the flow of blood and oxygen to the heart or to the brain, causing as a consequence, a heart attack or stroke, and can cause kidney damage, leading to kidney failure.

In studies by Mirzaeipour et al. in Iranian military personnel, hypertension was confirmed as an important risk factor of CVDs (O*R* = 1.641, *p* = 0.003) [9]. In studies by Gielerak et al., the increased prevalence of hypertension with age was revealed, as in 44.7% of Polish soldiers over 50 years of age, hypertension was reported. In over 50% of investigated soldiers, blood pressure was abnormally high when measured in the office, and 14% of the values corresponded to grade 2 or 3 hypertension. Furthermore, one third of normotensive soldiers had a high normal blood pressure. A total of 14% of soldiers reported a history of hypertension, and among them, the number of soldiers with increased blood pressure was particularly high (86%) [6]. 

Studies by Grosz et al. showed that high blood pressure affects 14.7% of Hungarian military pilots [19], whereas in Serbian military pilots, the prevalence of elevated blood pressure was 36.3% [13]. In 2007–2016, the rate of diagnosed hypertension in the total examined population of the US Army was 15.3, and it was 14.6 in the Air Force, whereas in pilots and crew, this rate was only 9.7. Similarly, as in Poland, hypertension incidence increased with advancing age, and the highest rates were found in those aged 50 years or older (rate of 54.4) [4]. 

Solovieva et al. compared blood pressures and heart rates in different flight phases (during takeoffs, in flight echelon, and during landings) in military pilots of Arctic transport in aviation suffering from essential hypertension and healthy pilots with CVD risk factors but without CVDs. They observed maximum blood pressure and heart rate values that were higher in hypertensive pilots than in the control subjects during each flight phase. The maximum systolic blood pressure during takeoff was 202 mmHg in the first group versus 179 mmHg in the control group. The highest heart rate during takeoff was also recorded in the first group in comparison to the control (164 beats/min versus 127 beats/min, respectively). The maximum systolic blood pressure and heart rate recorded during the landing in pilots of the first group were 253 mmHg and 150 beats/min, while the values in the control group were 163 mmHg and 141 beats/min, respectively. They concluded that military pilots with hypertension withstand the highest hemodynamic loads during flights in the harsh weather conditions of the Far North and can be permitted to operate airships in the presence of adequately controlled hypertension [15]. Moreover, Siagian et al., who observed hypertension in 49 of 549 examined Indonesian Air Force pilots and an almost two-fold risk of hypertension in helicopter pilots compared to pilots of fixed wing aircrafts (who are more exposed to noise that can reach 90–120 dB), confirmed that chronic noise exposure is a risk factor for blood hypertension. Chronic exposure to noise stimulates the sympathetic nervous and endocrine systems, which is reflected by a higher resting pulse rate and, as a consequence, a more than two times higher risk of hypertension. This was observed as an almost three times higher risk of hypertension in pilots with more than 1400 h of flying time [41,42].

### 4.2. Overweight and Obesity

The WHO defines overweight and obesity as abnormal or excessive fat accumulation that may impair health, based on body mass index (BMI), which relates to a person’s weight in kilograms divided by the square of his height in meters (kg/m^2^). A diagnosis of overweight is given to a person whose BMI is equal to or greater than 25, whereas a diagnosis of obese is given to a person whose BMI is equal or exceeding 30, despite the age or gender. The main cause of obesity and overweight is an energy imbalance between the calories consumed and the calories expended, resulting from an increased intake of energy-dense foods that are rich in fat and sugars and a decrease in physical activity as a consequence of sedentary work, changes in modes of transportation, or increased urbanization. The prevalence of obesity nearly tripled between 1975 and 2016. In 2016, according to WHO data, 39% of adults aged 18 years and over and 13% of the world’s adult population were overweight or obese, respectively [43]. Overweight and obesity are listed among risk factors not only of CDVs but also for numerous other noncommunicable diseases such as diabetes, osteoarthritis, and some cancers.

Obesity was the most common CVD risk factor diagnosed in the total examined population of the US Army, and its rate was 19.1 [4]. For the US Air Force, this rate was quite similar at 19.7, but in military pilots and crew, the obesity rate was significantly lower at only 8.1 [4]. Similarly, in Polish military pilots, the rate of obese soldiers was 6.6%, but over half (52.2%) of them were found to be mildly overweight [44]. Studies by Gielerak et al. revealed that 54.6% of the total investigated population of Polish soldiers was overweight, 14.1% of the total investigated population suffered from obesity, and the prevalence of both overweight and obesity increased with age and with a decrease in physical activity [6]. Overweight and obesity is a common problem among military pilots in Hungary. Grosz et al. revealed that 40.8% of Hungarian military pilots examined in 1994 suffered from obesity [19]. In studies of Radjen et al., a BMI of 25–30 kg/m^2^ (overweight) was observed in 58.1% of examined Serbian military pilots, while 16.2% of them were reported to have BMI > 30 kg/m^2^, which indicates obesity [13]. 

### 4.3. Diabetes

Diabetes, which is a chronic disease that occurs either when the pancreas does not produce enough insulin or when the body cannot effectively use the insulin it produces, affected 422 million of people in 2014, and its prevalence still increases, especially in low- and middle-income countries. Uncontrolled diabetes causes hyperglycemia, which over time, leads to serious damage to many of the body’s systems, especially the nerves (neuropathy), blood vessels (angiopathy), heart, eyes (retinopathy), and kidneys (nephropathy). According to the WHO, the risk of heart attack and stroke is two- to three-fold higher in diabetic patients in comparison with non-diabetic patients [45]. Blood glucose control is the first-choice test to detect the elevated level of glucose and to implement corrective actions such as body weight normalization, regular moderate-intensity physical activity, dietary intervention focused on the avoidance of sugar and saturated fats, and tobacco use avoidance. Most of these preventive actions are common for diabetes and CVDs.

The rate of abnormal glucose levels diagnosed in the total US Army population was 4.6, whereas the diabetes rate was only 0.8, and both rates increased with age in the total population. For the Air Force population, the values of both rates were similar to the general population, and for military pilots and crew, their values were slightly lower: 3.2 and 0.4, respectively [4]. However, in studies by Mirzaeipour et al., in Iranian military personnel, it was revealed that diabetes had the greatest impact (O*R* = 1.792, *p* = 0.005) on coronary artery disease risk among all examined risk factors [28]. In the entire examined population of Polish soldiers, the prevalence of previously diagnosed diabetes was rather low (1%). However it increased with age, reaching 7% of the examined soldiers over 50 years of age. The mean fasting glucose level in the total population was 88.8 ± 23.2 mg/dL, and it increased with the age, reaching 106.1 ± 53.7 mg/dL in the soldiers over 50 years of age. Plasma glucose concentrations exceeding 100 mg/dL were detected in 16% of all examined soldiers and in 43.5% of soldiers over 50 years of age [6]. Studies by Radjen et al. revealed fasting plasma glucose levels > 5.6 mmol/L in 50.3% of Serbian military pilots [13].

### 4.4. Hyperlipidemia

Hyperlipidemia refers to the pathological condition characterized by an elevated fasting total cholesterol level (hypercholesterolemia), which may or may not be accompanied by an elevated triglyceride level (hypertriglyceridemia) in the blood, which is recognized as a CVD risk factor [46]. A more objective definition describes hyperlipidemia as low-density lipoprotein (LDL-C), total cholesterol, triglyceride levels, or lipoprotein levels greater than the 90th percentile in comparison to the general population or an HDL-C level less than the 10th percentile when compared to the general population [47]. Both genetic and environmental factors are responsible for the appearance of hyperlipidemia, which results in hyperlipidemia’s subdivision into two subcategories: primary (familial) or secondary (acquired) hyperlipidemia. Primary hyperlipidemia derives from a plethora of inherited genetic disorders, whereas secondary hyperlipidemia typically originates from an alternate underlying etiology, such as an unhealthy diet, medications (amiodarone and glucocorticoids), hypothyroidism, uncontrolled diabetes, and/or a poor lifestyle regimen [47]. 

Disturbances in the lipid profile of the blood in airline pilots have been investigated as CVD risk factors for years [48]. Mirzaeipour et al. showed, in studies of military personnel in Iran, the positive association between hyperlipidemia and coronary artery disease (O*R* = 1.773, *p* = 0.002) [28]. Recent studies of Gielerak et al. showed that the total cholesterol level was increased in over half of examined Polish soldiers (52.1%), and the proportion of those with abnormal LDL-C was even higher (60%). Moreover, in 36.2% of soldiers, increased triglyceride levels were also diagnosed, and in 13% of soldiers, low HDL-C levels were observed [6]. Among Polish military pilots, hypercholesterolemia was diagnosed in 72.4%, and hypertriglyceridemia was diagnosed in 17.1%. Increased levels of LDL-C were found in 86.9% of military pilots, and in 69.9% of them, decreased levels of HDL-C were revealed [44]. Hyperlipidemia was recognized as the second most common CVD risk factor in the general population of the US Army, and its rate was 18.7. Its prevalence increased with age. In Air Forces, its rate was quite similar at 17.8. However, in military pilots and crews, the rate of hyperlipidemia was increased to 22.7. The annual incidence rate of hyperlipidemia decreased from 2007 to 2016 [4]. Grosz et al. reported elevated total cholesterol levels in 53.9% of Hungarian military pilots in 1994 [19]. Detailed studies by Radjen et al. on Serbian military pilots showed that total cholesterol > 5.2 mmol/L was found in 82.1% of subjects, and LDL-C > 3.38 mmol/L was found in 65.9% of the examined population. HDL-C < 1.00 mmol/L was detected in 27.9% of examined pilots, whereas a total cholesterol-to-HDL-C ratio > 6 was observed in 35.8% of them [13]. Moreover, Sammito et al. showed an increase with age in LDL-C levels in German military pilots [49]. 

### 4.5. Metabolic Syndrome

The coexistence of some of the aforementioned CVD risk factors is quite common and is defined as metabolic syndrome. Metabolic syndrome causes increased CVD risk and type 2 diabetes risk and a higher risk of death from all causes, including CVDs. Mottillo et al. revealed, that metabolic syndrome is associated with a two-fold increase in cardiovascular outcomes and a 1.5-fold increase in all-cause mortality [50]. What is of utmost importance is the fact that in metabolic syndrome the overall risk of CVDs exceeds the sum of the risks associated with each factor, which suggests hyperadditive synergy [13]. A meta-analysis performed by Mottillo et al. showed that metabolic syndrome was associated with an increased risk of CVDs (relative risk (RR): 2.35; 95% confidence interval (CI): 2.02 to 2.73), CVD mortality (RR: 2.40; 95% CI: 1.87 to 3.08), all-cause mortality (RR: 1.58; 95% CI: 1.39 to 1.78), myocardial infarction (RR: 1.99; 95% CI: 1.61 to 2.46), and stroke (RR: 2.27; 95% CI: 1.80 to 2.85). 

In studies by Weber et al. on German military pilots, metabolic syndrome was defined as systolic blood pressure > 130 mm Hg, cholesterol > 200 mg/dL, HDL < 40 mg/dL, triglycerides > 150 mg/dL, and fasting blood glucose > 100 mg/dL. The metabolic syndrome group consisted of 110 individuals meeting these criteria (0.9%, 95% CI 0.7–1.1%) whose BMI values were significantly higher than those of the healthy individuals and remained higher during the observational period. It should be emphasized that metabolic syndrome was significantly less prevalent in the examined population of military pilots than in the general population and was less prevalent than in other civil or military aviation samples. The prevalence of metabolic syndrome in military pilots in Germany increased significantly with the age of the individual participant (β = 0.06, *p* < 0.0001) but decreased with the duration of observation (β = −0.23, *p* < 0.0001). However, no relationship between the prevalence of metabolic syndrome and total flying hours was observed. In addition, no association between metabolic syndrome and work-related stress or shift work was observed. The authors claimed that military flying per se does not seem to contribute in a significant way to metabolic syndrome [18]. However, Radjen et al., who examined Serbian military pilots without CVDs, identified metabolic syndrome in 28.5% of them, wherein 49% of them had three components of metabolic syndrome and 29.4% of them had all five components of metabolic syndrome. This is a higher prevalence of metabolic syndrome than that observed for general adult European population (15%). Moreover, 72.1% of Serbian military pilots had increased carotid intima–media thickness [13], which is a valid marker of early and generalized atherosclerosis and increased CVDs risk [51]. Carotid intima–media thickness increased with the revealed number of metabolic syndrome components, and its independent predictors were total cholesterol and BMI [13]. Military pilots need to meet strict physical and health standards to be considered fit for flight. Due to the nature of their military service, they are periodically examined to assess their validity. Many authors claim that military pilots are rather healthy compared to the general military population and overall population [4,18]. However, studies by Grosz et al. revealed that CVDs represent the reason for approximately 10% of all groundings among military pilots in Hungary [19]. Moreover, Solovieva et al. revealed that in the Russian army the flight personnel found unfit for flight jobs at ages under 40 due to confirmed hypertension, atherosclerosis, chronic diseases of myocardium, and coronary arteries were more than 60% of the people found unfit due to visceral diseases [15]. Taking into account the time and resources spent on each military pilot, it is important to maintain their status, utility, and readiness for service. For this examination of different CVDs, risk is of the utmost importance, especially the early identification of risk factors and the implementation of remedial actions. 

## 5. Atherosclerosis

For many years atherosclerosis was considered an inevitable consequence of aging, and its importance as the substrate for morbidity and mortality in populations was poorly defined. This view started to change in mid-1940, and numerous studies led to the recognition of atherosclerosis as an inflammatory and immunologically driven response of the arterial wall to multifactorial repetitive injury [52]. Atherosclerosis begins at sites of endothelial injury. In atherosclerosis, plaques, which are made up of fat, cholesterol, calcium, and other substances found in the blood, build up in arteries, harden, and narrow the arteries, which limits the blood flow and oxygen supply. Common risk factors for atherosclerosis, which are hypercholesterolemia, diabetes, hypertension, tobacco smoking, aging, and nitrate intolerance, partially overlap the CVD risk factors, making atherosclerosis an intermediate in CVD development. Moreover, they increase the production of reactive oxygen species (ROS), not only by endothelial cells but also by vascular smooth muscle cells and adventitial cells, which initiate different processes involved in atherosclerosis initiation and progression [53]. It is evident that it is not actually atherosclerosis itself but the rupture of the atherosclerotic plaque, which exposes tissue factors and triggers clot formation, and in turn leads to the acute obstruction of the arterial lumen, that is responsible for most of the clinical events related to atherosclerosis [54]. 

## 6. Oxidative Stress

Oxidative stress is a pathological condition resulting from an imbalance between ROS formation and antioxidant defense mechanisms [55]. According to the theory of oxidative stress, atherosclerosis is the result of the oxidative modification of LDL-C by ROS in the arterial wall in the places of endothelial injury. Oxidative stress leads to the oxidation of LDL. The oxidative forms of LDL-C (oxLDL) are more easily taken up by macrophages, as they express scavenger receptors (e.g., CD36), which promote oxLDL internalization, and are involved in the formation of foam cells [55,56]. The lipid-laden macrophages are deposited underneath the endothelium of arteries, eventually forming obstructive atherosclerotic plaques [57]. OxLDL can induce many proatherogenic processes, including the modulation of oxidative burst [55]. ROS also initiate several other processes involved in the inception and development of atherosclerosis, such as the expression of adhesion molecules, the stimulation of vascular smooth muscle proliferation and migration, apoptosis in the endothelium, the oxidation of lipids, the activation of matrix metalloproteinases, and the alteration of vasomotor activity [53]. The reduced production and bioavailability of NO resulting from the increased production of ROS leads to vasoconstriction, platelet aggregation, and the adhesion of neutrophils to the endothelium [56]. Several enzyme systems contribute to the production of free radicals in the vessel wall: nicotinamide-adenine dinucleotide phosphate-oxidase (NAD(P)H oxidase), xanthine oxidase, nitric oxide synthase, myeloperoxidase, and lipoxygenases [56]. NAD(P)H oxidase and myeloperoxidase are used by macrophages to produce ROS and the myeloperoxidase pathway, especially, is responsible for oxidative damaging LDL in the artery wall. Lipid-laden macrophages are the hallmark of atherosclerotic lesions [54]. Oxidants generated by myeloperoxidase and metalloproteinases also play a role in triggering plaque rupture in the artery wall [54]. OxLDL may also induce other processes involved in plaque development, such as the maturation of dendritic cells and shifting from classical to alternative macrophage activation as well as from a T helper 1 to a T helper 2 response, which suggests the involvement of innate and adaptive immunity. Sublethal oxidative stress can activate redox-sensitive kinase cascades and transcription factors such as nuclear factor κB (NF-κB) and activating protein 1 (AP-1), with resulting increases in the inflammatory response. The antioxidant defenses of the body at the cellular level are mainly provided by different enzymes (SOD, catalase-CAT, GPx, and PON1) and glutathione (GSH), whereas in plasma, non-enzymatic antioxidants, such as metal-chelating antioxidants (transferrin, albumin, and ceruloplasmin), chain-breaking or free radical scavenging antioxidants (uric acid, bilirubin, thiols, vitamin E, ascorbic acid, and carotenoids), or urate, play the major role [55].

Flying conditions (high altitude and the low-pressure atmospheric oxygen) decrease oxygen availability, increase the formation of ROS and reactive nitrogen species (RNS), and deplete the antioxidant system. However, some adaptive mechanisms develop over time. Petraki et al. assessed the effects of a flight simulation on the antioxidative status (total antioxidant capacity—TAC, CAT, and GSH) as well as the oxidative modifications of proteins and lipids through the measurement of protein carbonyls (PCs) and thiobarbituric acid reactive species (TBARS) of military pilots with differing flying experiences. They observed an adaptation to the flight training in an effort to battle oxidative stress in experienced pilots, revealed by increased levels of GSH [58]. Corsi et al. proposed erythrocyte glycohydrolases as new sensitive markers to assess oxidative stress, and they investigated their concentration in air force pilots. They revealed better protection against oxidative stress in military pilots than in healthy civilian controls [59]. They claimed that supersonic aircraft pilots working at high altitude, even if exposed to physiological stresses, can, with proper diet, regular exercise, and periodical medical examinations, maintain a healthy balance between oxidant and antioxidant status [60]. Moreover, Zawadzka-Bartczak et al. confirmed higher activities of SOD and GPx and higher TAC in military pilots in comparison with men with advanced coronary atherosclerosis [61]. However, she also observed decreased activity of those antioxidant parameters in military pilots with elevated levels of total cholesterol and LDL-C, which confirms the involvement of oxidative stress in atherosclerosis pathogenesis [62,63]. 

## 7. Inflammation

Atherosclerosis is also considered a chronic inflammatory disease of the arterial wall, with a long asymptomatic phase, which initiates in the place of endothelial injury, where both innate and adaptive immune responses contribute to disease initiation and progression. Vascular inflammation, which is characterized by immune cell infiltration, contributes to atherosclerosis formation by the increased expression of adhesion molecules and their ligands, the extravasation of leukocytes, the activation of immune cells and pro-inflammatory signaling pathways, and an increase in oxidative stress and cytokine production in the arteries. The amplification of inflammation, which is mediated both by T-cell mediated immune responses and the humoral immune response, can lead to plaque rupture. Following this, thrombosis occurs when the thrombus is sufficiently large that it can either partially or completely occlude the coronary vessel lumen and cause an acute event [55]. Inflammation and oxidative stress are interrelated, as they form a vicious feedforward cycle during atherogenic plaque progress and thrombosis. On the one hand, oxidative stress activates transcription factors that alter inflammatory cytokines, soluble mediators, and chemokines. On the other hand, the cytokines and chemokines secreted by inflammatory cells gather inflammatory cells to the sites of inflammation, leading to increased ROS production [5,57]. It has been suggested that biomarkers of inflammation, such as increased blood homocysteine level, C-reactive protein (hs-CRP) level, and cytokine levels, should be considered and commonly studied as new significant predictive risk factors for future CVDs [5,64]. The Hs-CRP proinflammatory atherogenic marker is one of the independent predictors of cardiac disease. According to some authors, hs-CRP could be a valid and independent predictor of cardiac attack, cardiac arrest due to vascular obstruction, and vascular diseases. Mirzaii-Dizgah et al. revealed that the mean serum hs-CRP concentration significantly increased following the hypobaric hypoxia process (EHHP) in an altitude chamber (before: 1.78 ± 0.33 µg/mL vs. after: 2.58 ± 0.45 µg/mL) (*p* = 0.02) [65]. Due to this fact, hs-CRP should be considered as a useful biomarker in military pilots, as they experience hypoxia during flight. 

## 8. Genetic Background

It is estimated that 50% of patients suffering from coronary heart disease do not have the classical risk factors of hypercholesterolemia, hypertension, smoking, diabetes, obesity, or a sedentary lifestyle, which suggests the importance of genetic background of CVDs [64]. A family history of CVDs, which is frequently encountered in clinical practice, confirms the need for extensive studies of genetic background of CVDs. Many researchers emphasize that CVD appearance results from the complex synergistic reaction between environmental factors and genetic background, so the investigation of inherited gene variants, accompanied by the monitoring of environment factors, may be a better marker of individual CVD susceptibility. Genetic testing for risk loci associated with CVD may potentially improve clinical risk prediction, enable early pre-symptomatic or asymptomatic intervention, and improve the clinical management of CVDs.

Lusis et al. proposed a list of candidate genes that should be investigated to evaluate coronary heart disease risk [66]. For some of them, significant correlations have been proven.

The risk of coronary heart disease and myocardial infraction is increased by the appearance of autosomal dominant hypercholesterolemia, which occurs in three forms: (1) familiar hypercholesterolemia resulting from a mutation in the *LDLR* gene, (2) familial defective apolipoprotein B resulting from a mutation in the *APOB* gene, and (3) hypercholesterolemia, autosomal dominant, 3, which is caused by one of three missense mutations in the *PCSK9* gene. Familial combined hyperlipidemia, which manifests with elevated levels of very low density lipoprotein (VLDL), LDL, APOB, and triglycerides, seems to be related to the *APOA1/C3/A4/A5* gene cluster on chromosome 11. Familial hypertriglyceridemia with normal cholesterol levels but higher than normal triglyceride levels (>400 mg/dL) appears to be an autosomal dominant disorder that is connected with more than one locus linked to its transmission (15q11.2-q13.1 and 8q11-q13). Individuals with atherogenic lipoprotein phenotype (ALP) have a large number of small, dense, LDL (subclass pattern B) particles and triglyceride-rich lipoproteins and a decreased level of HDL, and this phenotype is linked to the LDLR locus, mapping to 19p13.3-p13.2 [67,68].

A number of studies have looked at the association between the *APOE* gene, encoding an LDL receptor ligand, apoE, and the risk of CHD, and a significant correlation between *APOE* Ɛ4 and coronary heart disease risk was established. Moreover, the *LPL* gene encoding lipoprotein lipase, an enzyme responsible for hydrolyzing triglyceride particles and indirectly responsible for the production of HDL-C, was investigated in relation to coronary heart disease, and the diversified influence of different mutations has been established [67,68].

In 2007, three GWA studies simultaneously reported a locus at chromosome 9p21.3 that was significantly associated with coronary artery disease. At this locus, *ANRIL*, which is a large non-coding RNA of unknown function that was revealed to be expressed in the vascular endothelium, monocyte-derived macrophages and coronary smooth muscle cells, and two protein-coding genes, *CDKN2A* and *CDKN2B*, encoding cyclin-dependent kinase inhibitors, were found [69,70]. Four single-nucleotide polymorphisms (SNPs) on chromosome 9p21 associated with CAD (rs10757274 and rs2383206) and myocardial infarction (MI: rs2383207 and rs10757278) in White populations in Northern Europe and North America were identified. The same SNPs were confirmed to be associated with coronary artery disease in the population of South Korea [71]. However, coronary artery disease susceptibility is considered to be influenced by many gene polymorphisms and gene–gene interactions. Among them, the *PPARG* gene, which is located at 3p25-24 and plays a role in adipocyte differentiation, insulin sensitivity regulation, fatty acid metabolism regulation, adipose tissue storage, and free fatty acid reduction, and the *CYP1A1* gene, which mediates the oxidative metabolism of exogenous and endogenous molecules, e.g., cholesterol, estrogens, or androgens, are both risk factors of coronary artery disease. Zhang et al. revealed in the Chinese Han population that variants in rs10865710, rs1805192, and rs4646903 were associated with higher coronary artery disease risk. They also found a significant interaction between rs1805192 and rs4646903. The risk of coronary artery disease was the highest in participants with rs1805192- Pro/Ala or Ala/Ala and the rs4646903- TC+CC genotype and was the lowest in those with rs1805192- Pro/Pro and the rs4646903- TT genotype [72].

Moreover, some genes involved in the regulation of oxidative stress and the risk of atherosclerosis have been studied to associate the influence of their polymorphism on oxidative stress. Katakami et al. investigated four relatively common genetic variants related to oxidative stress (*GCLM C-588T* (rs41303970), *MPO G-463A* (rs2333227), *PON1 Gln192Arg* (rs662), and *NAD(P)H oxidase p22phox C242T* (rs4673)) and their influence on atherosclerosis. They revealed that the GCLM C-588T polymorphism was associated with elevated atherosclerosis risk (*r =* 0.090, *p* = 0.0008), but the associations between the other three polymorphisms did not reach statistical significance. Moreover, they observed a synergistic effect of all investigated polymorphisms, as the atherogenesis risk was significantly greater as the total number of the four concomitant ‘‘pro-oxidant alleles” in each subject was increased (*r =* 0.108, *p* < 0.0001). Furthermore, the number of ‘‘pro-oxidant alleles” was a risk factor for a high atherosclerosis risk independent of conventional risk factors (*p* = 0.0003) [73]. 

Selected genes with immune regulatory functions are part of the complex genetic background contributing to inflammation and atherosclerosis initiation and progression as well as CVDs. Ianni et al. investigated the genetic variations (SNPs) in the promoter region of a number of genes regulating metabolic and immune functions (*VEGF*, *ACT*, *HMGCR*, *IL-1β*, *IL-10,* and *IFN-γ* genes) that were previously identified as being associated with an increased risk of CVDs. They revealed that the concomitant presence of the CC genotype of *VEGF*, the A allele of *IL-10,* and the A allele of *IFN-γ* genes was associated with an increased risk of acute myocardial infarction and was more frequent among patients with a positive parental history of acute myocardial infarction, which confirmed that these genes with immune regulatory functions are part of the complex genetic background contributing to familiarity for CVDs [64]. The association between the Asp299Gly (+896A>G; rs4986790) polymorphism in the *TLR-4* gene and atherosclerosis risk has been proven by the detection of lower levels of pro-inflammatory IL-6 and TNF-α and higher levels of anti-inflammatory IL-10 in carriers bearing the *TLR-4* polymorphism. The CD14 gene -260C>T polymorphism has been established as risk factor of coronary heart disease, especially in East Asians. The *ICAM-1* gene -469E>K polymorphism was proven to be significantly associated with an increased risk of coronary heart disease in Asian and Caucasian populations. In the case of the *TNF-α* gene, a pooled analysis revealed that carrying the *TNF-α* gene A variant conferred a 1.5-fold increased risk of developing coronary heart disease in the Caucasian population. Moreover, the *MCP* gene -2518A>G SNP, known to increase MCP-1 production, showed an association with an increased risk of CVD in Caucasians [5]. Pollard et al., who hypothesized that there might be a quantifiable genetic basis for the linkage of post-traumatic stress disorder (PTSD) with the increased propensity for CVDs in soldiers, found 37 of the PTSD candidate risk genes that are also candidate independent risk genes for CVDs. Their analysis revealed the innate immunity and NF kB-mediated inflammation as a possible mechanism for this linkage [74]. Zhao et al. showed that the expression change of eight genes (*NME4*, *PNPLA7*, *GGT5*, *PTGS2*, *IGF1R*, *NT5C2*, *ENTPD1,* and *PTEN*) could be a sign of elevation of blood pressure for military pilots [75]. 

## 9. Sex Hormones

Among CVD risk factors in both men and women, the levels of sex hormones are considered to be meaningful. Among post-menopausal women, a higher testosterone/estradiol ratio was associated with an elevated risk for CVD incidents and coronary heart disease (CHD), and higher levels of testosterone were associated with increased CVD and CHD, whereas higher estradiol levels were associated with a lower CHD risk [76]. However, prospective studies by Barrett-Connor et al. did not reveal a causal or preventive role for endogenous estrogens or androgens and cardiovascular mortality in elderly women [77]. In men, testosterone, dihydrotestosterone, dehydroepiandrosterone (DHEA) and its sulfate (DHEA-S) androstenedione, and estradiol are classified as sex hormones. Cross-sectional studies have found high testosterone levels to be associated with high HDL-C levels, low LDL-C, and low triglyceride levels [78]. Epidemiological data show that low total testosterone levels in men are associated with a higher risk of CVDs due to their influence on CVD endogenous risk factors and mortality. Khazai et al. confirmed the positive correlation between low testosterone levels and coronary atherosclerosis [35]. However, Srinath et al. concluded that neither high nor low testosterone levels directly predict atherosclerosis. Nevertheless, they confirmed testosterone levels as markers for other cardiovascular risk factors, as they observed significant dependencies between low testosterone levels and BMI, greater waist circumference, diabetes appearance, hypertension, and lower HDL-C levels (*p* = 0.01) [79]. One of the proposed explanations of testosterone activity is its direct influence on HDL-C levels, resulting from the increase in the hepatic production of apolipoprotein A-I, which is the major protein constituent of nascent HDL particles. Steroid hormones may also act via androgen receptors, which are located in systemic arteries (including the aortic, coronary, pulmonary, and carotid arteries) and modulate smooth muscle tone. Testosterone may also exert its vasodilatory effect via an interaction with potassium and calcium [78]. 

## 10. Conclusions

A number of findings confirm that CVD risk may be diminished by the elimination or limitation of modifiable risk factors. Although among soldiers the risk of CVDs is lower than in general population, the identification of the early stages of developing CVDs is currently limited. For a correct and early estimation of CVD risk in asymptomatic soldiers, especially if no environmental risk factors coexist, the scope of the performed tests should be increased with not only additional blood indicators related to oxidative stress, inflammation, or hormonal status but also with genetic studies. The indication of risk groups among military professionals, especially military pilots, enables the implementation of early preventive activities, which will prolong the state of health and military suitability of this professional group.

## Data Availability

Not applicable.
